# *NTN1* Affects Porcine Intramuscular Fat Content by Affecting the Expression of Myogenic Regulatory Factors

**DOI:** 10.3390/ani9090609

**Published:** 2019-08-27

**Authors:** Ligang Wang, Lingling Zhao, Longchao Zhang, Xin Liu, Xinhua Hou, Hongmei Gao, Hua Yan, Fuping Zhao, Lixian Wang

**Affiliations:** 1 Institute of Animal Science, Chinese Academy of Agricultural Sciences, Beijing 100193, China; 2 College of Animal Science and Technology, Qingdao Agricultural University, Qingdao 266109, China

**Keywords:** copy number variation, intramuscular fat content, *NTN1*, swine

## Abstract

**Simple Summary:**

Intramuscular fat (IMF) is a key meat quality trait in the pork industry. In this study, we validated the effect of the copy number of Netrin-1 (NTN1-CNV) on Netrin-1 (NTN1) protein expression and explored the possible affective mechanism of *NTN1* on IMF. The results indicated that NTN1-CNV may affect the expression of NTN1 protein by its gene dose, and the expression of *NTN1* may affect the proliferation and differentiation of muscle cells by the AMP-activated protein kinase (AMPK) pathway and finally influence the IMF content.

**Abstract:**

Intramuscular fat (IMF) content is an important economic trait for pork quality. Our previous results regarding the genome-wide association between IMF content and copy number variations (CNVs) indicated that the CNV within Netrin-1(NTN1-CNV) was significantly associated with IMF. In order to validate the effect of NTN1-CNV, we detected the Netrin-1 (*NTN1*) gene dose and protein expression content in the longissimus dorsi of different IMF content pigs using Western blotting and investigated the expression of *NTN1* RNA in different tissues using real-time quantitative polymerase chain reaction (qPCR). The knock-down of the *NTN1* gene in C2C12 and 3T3-L1 cells and over-expression in C2C12 cells during the proliferation and differentiation stage were also investigated to explore the possible pathway of action of *NTN1.* The results showed that in individuals with IMF content differences, the gene dose of NTN1 and the expression of NTN1 protein were also significantly different, which indicated that NTN1-CNV may directly affect IMF by its coding protein. *NTN1* had the highest expression in pig longissimus dorsi and backfat tissues, which indicates that *NTN1* may play an important role in muscle and fat tissues. The in vitro validation assay indicated that *NTN1* silencing could promote the proliferation and inhibit the differentiation of C2C12 cells, with no effect on 3T3-L1 cells. Additionally, *NTN1* over-expression could inhibit the proliferation and promote the differentiation of C2C12 cells. Combined with previous research, we conclude that NTN1-CNV may affect IMF by its gene dose, and the expression of *NTN1* may affect the proliferation and differentiation of muscle cells by the AMP-activated protein kinase (AMPK) pathway and finally influence the IMF.

## 1. Introduction

In the past few decades, with the continuous breeding of pig litter sizes, growth rate, back-fat thickness and other traits, the quality of pork has been declining [1]. Intramuscular fat (IMF) content is an important factor affecting pork quality. The content of IMF is closely related to the flavor, tenderness and juiciness of pork. A suitable intramuscular fat content can produce a better taste and is an important factor in determining meat quality [2].

As one of the most extensive variations of the genome, copy number variations (CNVs) play an important role in many traits and are the most likely mutations to explain the “missing inheritance” beyond the single nucleotide polymorphism (SNP) effect [3]. In animals, especially in pigs, research into CNV maps and functions has made many important advances. In 2008, using array-based comparative genomic hybridization (aCGH) technology, 13 pigs were selected as experimental materials to construct maps of 37 CNV regions on chromosomes 4, 7 and 17 of pigs [4]. From 2014, the SNP chip and next generation sequencing (NGS) were used to detect CNV in different Chinese and foreign pig breeds, and a total number of 4145 CNVRs were detected [5,6,7,8,9]. In one of the global analyses of CNVs in pigs using sequencing data, one CNV in the gene glycerol-3-phosphate acyltransferase 2 (*GPAT2*) was found to be significantly associated with the C18:2(n-6)/C18:3(n-3) ratio in backfat and carcass length [5]. In the research of Ran et al. (2018), copy number variations of the methenyltetrahydrofolate synthetase domain containing (*MTHFSD*) gene was significantly associated with litter size traits in Xiang pigs [6]. In one study on 3892 pigs, Stafuzza et al. found that four CNV regions on SSC2 (4.2–5.2 Mb), SSC3 (3.9–4.9 Mb), SSC12 (56.6–57.6 Mb) and SSC17 (17.3–18.3 Mb) were associated with the number of piglets born alive [7].

We previously performed a genome-wide association study of IMF traits on the F2 generation phenotypic data of large white ×Min pigs and found that the CNV (on chromosome 12, from 56,893,678 bp to 57,020,468 bp) within Netrin-1 (*NTN1*, also called *UNC-6*, on chromosome 12, from 56,822,597 bp to 57,056,391 bp) was significantly associated with IMF [8]. In previous research, we also found that DNA dosages were consistent with NTN1 RNA expression, and the NTN1 RNA expression was consistent with IMF content. *NTN1* is a member of the laminin-associated secreted protein family, and its function is currently unclear. Studies in humans and mice have shown that the *NTN1* gene is involved in a variety of cellular function-related metabolic pathways and is associated with the extension of synapses and cell migration, apoptosis, and differentiation during development [10,11]. Studies in 2014 showed that *NTN1* gene expression was significantly higher in human and murine obese fat cells than in lean fat cells [12]. The Adenosine a2b receptor (a2bR) is one of the receptors which NTN1 protein could bind directly to [13]. A previous study showed that the *a2bR* gene was involved in some well-known fat deposition pathways such as Cyclic adenosine monophosphate (cAMP)-dependent protein kinase (cAMP-PKA), the mitogen-activated protein kinase (MAPK) pathway, and so on [14]. Although there are many studies on the *NTN1* gene in humans and mice, beyond our research, there is no report that their CNV has any influence on traits. This study aimed to confirm the effect of the *NTN1* gene and its internal copy number variation (NTN1-CNV) on muscle and fat.

## 2. Materials and Methods

### 2.1. Ethics Statements

All methods and procedures in our study were carried out according to the standard guidelines on experimental animals, which were established by the Animal Ethics Committee of the Institute of Animal Science, Chinese Academy of Agricultural Sciences (IAS-CAAS) (Beijing, China). The experimental protocols were approved by the Science Research Department of IAS-CAAS (No. IASCAAS-AE-09).

### 2.2. Sample Collection

In order to investigate the *NTN1* RNA expression in different tissues, the heart, liver, spleen, kidney, longissimus dorsi and backfat were obtained from five individuals in the F2 resource population [8] who were randomly selected. The F2 resource population was constructed using Large White and Min pigs as F0 generation pigs, and the population size was 602 individuals. Pigs were weighed and sacrificed at 240 ± 7 days following standard commercial procedures. To detect the gene dose of *NTN1* and NTN1 protein expression, nine pigs with high (IMF content > 5.0) and low (IMF content < 1.5) IMF were selected for longissimus dorsi collection. IMF contents were measured using an ether extraction method (Soxtec Avanti 2055 Fat Extraction System, Foss Tecator, Hilleroed, Denmark). All samples collected were placed in a −80 °C freezer for the later extraction of tissue RNA and protein extraction.

### 2.3. Western Blotting Analysis

To detect the protein expression of NTN1 and adenosine, the longissimus dorsi of 5 high-IMF and 4 low-IMF individuals were selected to extract total protein. Total proteins were isolated in radioimmunoprecipitation assay (RIPA) lysis buffer (Beijing, China) with phenylmethylsulphonyl fluoride (PMSF), and the protein content was determined by a bicinchoninic acid (BCA) protein Quantitation Kit (Thermo Fisher Scientific, Waltham, MA, USA). Equal amounts of protein, with SDS-PAGE separation, were transferred to the polyvinylidene fluoride membranes. The membrane was sealed with 5% non-fat milk at room temperature for 2 h, incubated at 4 °C overnight, and the primary hybridized antibody was washed three times with tris-buffered saline and tween 20 (TBST). The secondary antibody was hybridized and washed again. Finally, the immunoblotting was visualized by an enhanced chemiluminescence (ECL) kit, and the optical density of the target band was analyzed by Quantity One image analysis software. Protein expression was normalized to β-actin expression.

### 2.4. Gene Dose of NTN1 in Individuals with Different Intramuscular Fat, and NTN1 RNA Expression in Different Tissues

To detect the gene dose of *NTN1* in individuals with different IMF and *NTN1* RNA expression in different tissues, real-time quantitative PCR amplification was performed using a SYBR^®^ green kit (TaKaRa Bio, Shiga Prefectur, Japan) on a Bio-Rad IQTM5 system (Bio-Rad, Hercules, CA, USA). When detecting the gene dose of *NTN1*, the glucagon gene (*GCG*) was used as a single copy control. The copy number was calculated by the method of 2^−ΔΔCT^, where Δ CT was the differential value of the target region cycle threshold (CT) and of the control region CT. Moreover, 2^−ΔΔCT^ stands for the comparison of the Δ CT value of samples with CNV to those without CNV. When detecting the *NTN1* RNA expression in different tissues, glyceraldehyde-3-phosphate dehydrogenase (*GAPDH*) was used as an internal reference, and the expression level of the gene was also calculated by 2^−△△CT^. A list of primer sequences is shown in Table 1.

### 2.5. NTN1 RNA Silencing in C2C12 Cells

To investigate the role of *NTN1* in muscle cell proliferation and differentiation, si-RNA was used to interfere with the expression of C2C12 cells first. The C2C12 cells were cultured in Dulbecco’s modified Eagle medium (DMEM, Gibico, Gaithersburg, MD, USA) containing 10% fetal bovine serum (FBS, Gibico, Gaithersburg, MD, USA) and 1% penicillin streptomycin (PS, Gibico, Gaithersburg, MD, USA) at 37 °C under a 5% CO_2_ atmosphere. Three *NTN1* si-RNAs and one negative control (NC) were designed in GenePharma (Table 2), transfected at a cell density of 70%, and the transfected reagent lipofectamine^®^ 2000 (Invitrogen, Carlsbad, CA, USA) was mixed with si-RNA and NC according to the instructions. The Opti-MEM medium (Gibico, Gaithersburg, MD, USA) was replaced with DMEM medium (Gibico, Gaithersburg, MD, USA) for 5 h. The complete medium was changed to the differentiation medium (including DMEM supplemented with 2% horse serum (Sigma, Ronkonkoma, NY, USA) and 1% PS solution) after continuous culturing for 24 h (defined on day 0, proliferative phase). After the differentiation medium was changed, cells were fused to 90% (defined day 0); then, the liquid was changed every day until day 3. Cells on day 0, day 1, day 2 and day 3 were collected for RNA extraction after induction. Each set of experiments was set up with three independent replicates.

### 2.6. RNA Silencing in 3T3-L1 Pre-Adipocytes Cells

To investigate the role of *NTN1* in fat cell proliferation and differentiation, si-RNA was used to interfere with the expression in 3T3-L1 cells (Tianwei, Beijing, China). The culture of 3T3-L1 pre-adipocytes cells was carried out in accordance with the above C2C12 culture. The transfection of *NTN1* si-RNA and NC was also carried out in accordance with the above C2C12 transfection. The complete medium was changed to the differentiation medium after continuous culture for 48 h (defined day 0, proliferative phase). The differentiation of 3T3-L1 cells was induced two days after fusion in DMEM containing 0.5 mM 1-methy1-3-isobutylxanthine (IBMX, Sigma, USA), 1 μM dexamethasone (DEX, Sigma, USA) and 5 μg/mL insulin. After two days (day 2), the maintenance solution (10 μg/mL insulin complete medium) was replaced and the culture was continued. The liquid was changed every day until day 8. Cells on day 2, 4, 6 and 8 were collected for RNA extraction after induction. After 6 days of differentiation of the adipocytes, oil red O staining and the detection of the accumulation of plasmid were performed. 3T3-L1 cells were washed three times with PBS, and then fixed with 4% formaldehyde fixative at 37 °C for 45 min. After repeating the washing three times, they were stained with oil red O for 2 h, the stain solution was discarded and the floating color was washed. The treated cells were observed under an inverted microscope (Tokyo, Japan).

### 2.7. RNA Over-Expression in C2C12 Cells

To investigate the role of *NTN1* in muscle cell proliferation and differentiation, RNA over-expression was also used in C2C12 cells (Tianwei, Beijing, China). One over-expression plasmid, named PEX-3 (pGCMV/MCS/Neo), and one negative control named PEX-1 (pGCMV/MCS/EGFP/Neo) with the binding site of XhoI/EcoRI was designed and synthesized by GenePharma. After the procedures of amplification, transformation and plasmid extraction, pEX-3 and PEX-1 were transfected in C2C12 at a cell density of 70%. The procedure of cell proliferation and differentiation was the same as the aforementioned.

### 2.8. Real-Time Quantitative Polymerase Chain Reaction (qPCR) Assay

The different expressions of si-RNA and regulating factors during cell proliferation and differentiation were detected using the qPCR method. Cyclin-dependent kinase inhibitor (*P21*), antigen identified by monoclonal antibody Ki 67 (*KI67*), muscle creatine kinase (*MCK*), myogenin (*MYOG*) and class I myosin (*MYOD*) were selected as regulator factors in muscle cell proliferation and differentiation. *Cyclin D1*, cyclin dependent kinase 4 (*CDK4*), peroxisome proliferator-activated receptor gamma (*PPARγ*) and fatty acid binding protein 4 (*FABP4*) were selected as regulating factors in adipose proliferation and differentiation. The total RNA of the harvest cells was extracted using Trizol^®^ reagent. The reverse transcription of RNA into single-stranded DNA was performed using a reverse transcription kit. Real-time quantitative PCR was performed using a SYBR^®^ green kit (TaKaRa Bio) on a Bio-Rad IQTM5 system (Bio-Rad, Hercules, CA, USA). GAPDH was used as an internal reference, and the expression level of the gene was calculated by 2^−△△CT^. The primers of the remaining genes are shown in Table 1.

### 2.9. Statistical Analysis

The Tukey HSD test was used for comparison across multiple tissues, and a t-test was performed to evaluate the statistical significance of the two-part comparisons of expression difference. In vitro, there were three replicates in each group. Statistical analysis was performed using SAS 9.4 statistical software (SAS Institute, Cary, CA, USA). A difference of *p* < 0.05 was considered to be statistically significant.

## 3. Results

### 3.1. Expression of NTN1 Protein in Longissimus Dorsi with Different Intramuscular Fat

**Figure 1:**
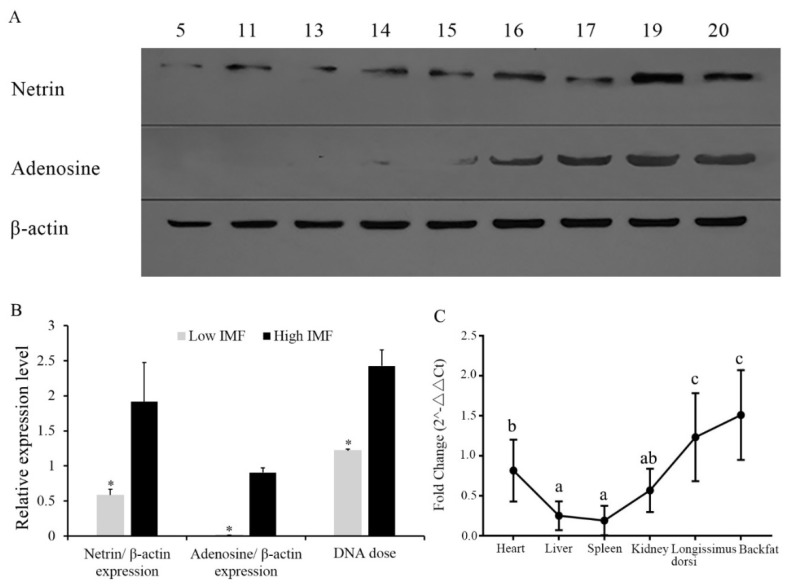
DNA, RNA, and protein expression of NTN1. (**A**) Protein expression of netrin and adenosine (n = 9, individual 5, 11, 13, 14 and 15 were low Intramuscular Fat (IMF), and individuals 16, 17, 19 and 20 were high IMF.). (**B**) Differences of NTN1 and adenosine protein expression and *NTN1* gene dose in high and low IMF pig longissimus dorsi. (**C**) Expression of *NTN1* gene in different tissues. * *p* < 0.05.

Individuals 5, 11, 13, 14 and 15 were selected from the low-IMF group, and the individuals 16, 17, 19 and 20 were selected from the high-IMF group. In Figure 1, it can be observed that the expression of netrin and the downstream protein adenosine in the high-IMF group was significantly higher than that of the low IMF group (Figure 1A,B).

### 3.2. Gene Dose of NTN1 in Individuals with Different Intramuscular Fat, and NTN1 RNA Expression in Different Tissues

From Figure 1B, the gene dose of *NTN1* in high-IMF individuals were significantly higher than in low-IMF individuals. The results (Figure 1C) showed that the *NTN1* expression level in the longissimus dorsi, backfat and heart were significantly higher than in other tissues (*p* < 0.05). The *NTN1* gene had the highest expression in the longissimus dorsi and backfat, followed by the heart and kidney, and the lowest expression in the liver and spleen. As C2C12 and 3T3-L1 cell lines are commonly the cell lines used in biological research into muscle and adipose tissue, we chose these two cell lines to perform the following research.

### 3.3. Effect of si-NTN1 on the Proliferation and Differentiation of C2C12 Cells

**Figure 2:**
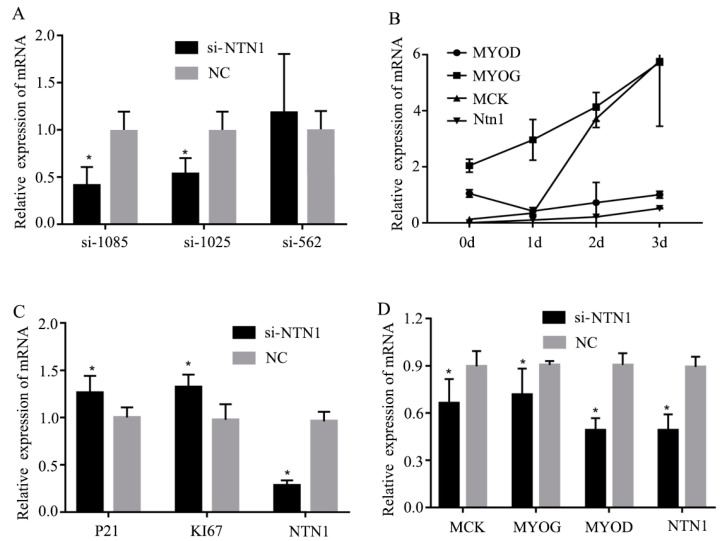
Effect of si-*NTN1* on the proliferation and differentiation of C2C12 cells. (**A**) The interference efficiency of three *NTN1* si-RNAs. (**B**) Expression of *NTN1,* antigen identified by monoclonal antibody Ki 67(*KI67*), and cyclin-dependent kinase inhibitor (*P21*) gene in C2C12 cell proliferation phase. (**C**) The expression levels of *NTN1* and muscle creatine kinase (*MCK*)*,* myogenin (*MYOG*) and class I myosin (*MYOD*) in normal differentiation C2C12 cells. (**D**) Expression of muscle creatine kinase (*MCK*), *MYOG*, *MYOD*, and *NTN1* gene in C2C12 cell differentiation phase. The results were represented as mean ± SD. The expression level of genes in si-*NTN1* C2C12 cells was determined by q-PCR using NC as the control. * *p* < 0.05.

Three si-NTN1s named si-1225, si-562 and si-1085, were transfected into C2C12 cells and assayed for *NTN1* interference efficiency testing. The results showed that in si-1085 and si-1025 interference cells, the expression of *NTN1* RNA were significantly different from NC interference cells (Figure 2A, *p* < 0.05), which indicated good transfection efficiency. Finally, si-1085 was selected to mirror the subsequent experiments.

As shown in Figure 2B, during the period of proliferation, when *NTN1* was successfully silenced, the expression of cyclin-dependent kinase inhibitor (*P21*) significantly decreased and the expression of the antigen identified by the monoclonal antibody Ki 67 (*KI67*) significantly increased. As shown in Figure 2C, in the normal differentiation stage, the expression of *NTN1,* muscle creatine kinase *(MCK),* myogenin (*MYOG*) and class I myosin (*MYOD)* on day 3 were higher than on other days. Thus, the expression of these genes at 72 h after transfection were detected. As shown in Figure 2D, when the *NTN1* was silenced, the expression levels of *MCK*, *MYOG* and *MYOD* also significantly decreased.

### 3.4. Effect of Over-Expression of NTN1 on the Proliferation and Differentiation of C2C12 Cells

**Figure 3:**
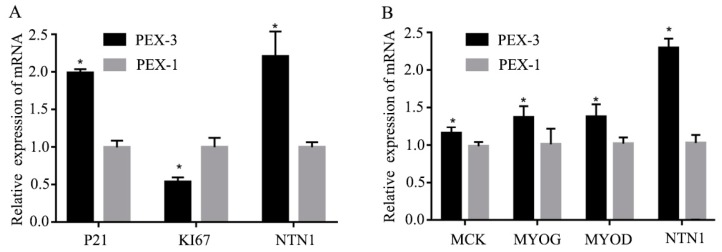
Effect of over-expression of *NTN1* on the proliferation and differentiation of C2C12 cells. (**A**) Expression of *NTN1*, *KI67*, and *P21* gene in C2C12 cell proliferation phase. (**B**) Expression of *MCK, MYOG, MYOD*, and *NTN1* gene in C2C12 cell differentiation phase. The results were represented as mean ± SD. The expression level of genes in *NTN1* over-expression C2C12 cells was determined by q-PCR using NC as the control. PEX-3 and PEX-1 were the over-expression plasmid, and the negative control, respectively. * *p* < 0.05.

*NTN1* over-expression was also used to investigate the role of *NTN1* in muscle cell proliferation and differentiation. As shown in Figure 3A, when *NTN1* was over expressed, the expression of *P21* significantly increased and the expression of *KI67* significantly decreased. As shown in Figure 3B, when the *NTN1* was over-expressed, the expression levels of *MCK*, *MYOG* and *MYOD* also significantly increased.

### 3.5. Effect of si-NTN1 on the Proliferation and Differentiation of 3T3-L1 Cells

**Figure 4:**
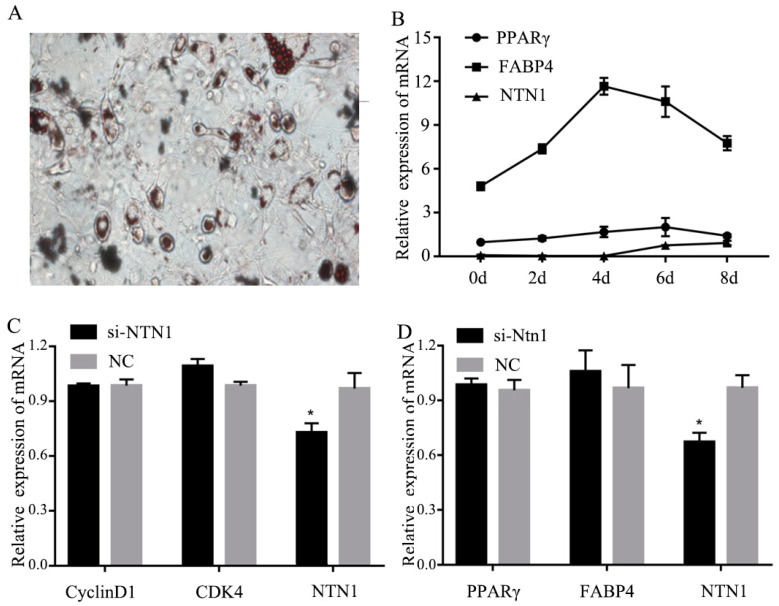
Effect of si-*NTN1* on the proliferation and differentiation of 3T3-L1 cells. (**A**) Oil red O staining of differentiation 3T3-L1 cells. (**B**) The expression levels of *NTN1,* Peroxisome proliferator-activated receptor gamma *(PPARγ)* and fatty acid binding protein 4 (*FABP4*) in 3T3-L1 adipocytes. (**C**) Expression of *Cyclin D1*, cyclin dependent kinase 4 (*CDK4*) and *NTN1* gene in 3T3-L1 cell proliferation phase. (**D**) Expression of P*PARγ*, *FABP4*, and *NTN1* gene in 3T3-L1 cell differentiation phase. The results were represented as mean ± SD. The expression level of genes in si-*NTN1* 3T3-L1 cells was determined by q-PCR using NC as the control. * *p* < 0.05.

The results showed that the expression of *NTN1* significantly decreased after the transfection of si-NTN1 in the proliferative phase, while the expression of genes related to adipose proliferation such as Cyclin D1 and cyclin dependent kinase 4 (*CDK4*), showed no difference compared to NC cells (Figure 4C). After transfection and differentiation, day-6 cells were collected and stained with Oil red O, and fat particles were observable. From Figure 4A,B, we can see the 3T3-L1 precursor adipocytes were well differentiated on day 6. The expression levels of *NTN1,* peroxisome proliferator-activated receptor gamma *(PPARγ*) and fatty acid binding protein 4 (*FABP4*) genes were not significantly different. Therefore, NTN1 may not have had an effect during the proliferation and differentiation of fat (Figure 4D).

## 4. Discussion

IMF deposition is a dynamic process that is regulated comprehensively by many factors such as hormones and cell factors [15]. The regulation of IMF deposition proceeds by a complex pathway, which interacts with muscle, fat and connective tissue [16].

*NTN1* is a protein-coding gene, which is involved in many biological processes. In 2014, studies confirmed that the expression of the *NTN1* gene in human and mouse obese fat cells was significantly higher than that of lean fat cells [11]. Among the genes of the NTN1 interaction protein, fibroblast growth factor receptor 1 (*FGFR1*) and v-myb myeloblastosis viral oncogene homolog (*MYB*), which interacts with *a2bR* and *NTN1,* were found to be different in muscles with different IMF contents [17]. In this study, the detection of netrin and adenosine protein expression revealed that NTN1 protein was significantly different in high and low-IMF content pigs. Additionally, the expressions of netrin and adenosine were identical, which was consistent with the results of previous research [18]. In our previous research, *NTN1* RNA expression was consistent with the copy number variation within *NTN1* [8]; in this study, we found that both the *NTN1* gene dose and NTN1 protein expression were consistent with IMF content, and we confirmed that NTN1-CNV affects IMF content by the gene dose.

The tissue expression profile results show that *NTN1* may be involved in the development of muscle and fat in pigs. IMF deposition was determined not only by adipogenic but also fibrogenic processes [19,20], and this may explain why *NTN1* has the highest expression in longissimus dorsi and backfat. The expression of *NTN1* in the heart is also very high, and this may relate to the *NTN1* function of cardiac protection [21]. However, in order to research how *NTN1* affects muscle and fat, we chose the C2C12 myoblasts and 3T3-L1 precursor adipocytes to perform in vitro validation.

In the process of muscle fiber formation, myoblast proliferation is a key stage in the early stage of muscle, which is regulated by a variety of signaling factors. *P21*, which is a cell cycle inhibitor, is one of the famous regulating factors, which can inhibit myoblast proliferation [22]. KI67 is another famous myoblast proliferation-associated protein [23]. Thus, we chose these two genes to validate the effect of *NTN1* on myoblast proliferation. The silencing and over-expression of *NTN1* could affect the expression of both *P21* and *KI67*, and this result indicates that the duplication of NTN1-CNV may inhibit myoblast proliferation. When myoblasts enter the differentiation stage, many regulatory factors are present, including myogenic regulatory factor (MRFs). MRFs are part of the transcription factor superfamily, including *MYOD*, *MYOG*, myogenic factor 5 (*MYf5*) and myogenic regulatory factor 4 (*MRF4*), which can stimulate the expression of myoblast differentiation. With the differentiation of myoblasts, the expression of *MYf5* and *MYOD* increased gradually [24]. Furthermore, *MCK* is a late differentiation stage marker [25]. When we knocked-down *NTN1* expression, *MYOG*, *MYOD* and *MCK* expression levels decreased in the differentiation phase compared with the control NC. When we over-expressed *NTN1*, the expressions of *MYOG*, *MYOD* and *MCK* increased consistently. This result has indicated that the duplication of NTN1-CNV may play an important role in promoting myoblast differentiation.

In previous research, *P21*, *KI67*, *MYOD*, *MCK* and *NTN1* has been shown to be involved in the AMP-activated protein kinase (AMPK)-related pathway in different tissues [25,26,27,28,29], and the AMPK pathway has recently been proven to be associated with IMF content in beef and pigs [30]. We inferred that *NTN1* may affect the IMF content by an AMPK-related pathway.

Precursor fat cells can only become mature fat cells to form fat after proliferating and differentiating. The cell proliferation cycle is divided into G1, S and G2 phases to regulate cell proliferation. In the proliferation stage, the expression of *Cyclin D1* and *CDK4* is usually high in adipocytes [31,32]. In the differentiation stage, the activation of *PPARγ* and *FABP4* could stimulate adipogenesis in fat cells [33,34]. In order to investigate the effect of *NTN1* on adipocyte proliferation and differentiation, we silenced *NTN1* in adipocytes and then detected the expression level of *CyclinD1*, *CDK4*, *PPARγ* and *FABP4*. We found that there was no different expression compared to the NC cells. *PPARγ* plays an important role in the differentiation process of adipocytes. In some research, *PPARγ* mRNA expression was significantly and positively correlated with IMF deposition (*p* < 0.05) [35]. Additionally, it was found that NTN1 protein interacted with a2bR, and studies by cell lines indicated that the overexpression of *a2bR* could inhibit fat production and regeneration [13,17]. However, in our research, we could not detect the effect of *NTN1* on *CyclinD1*, *CDK4*, *PPARγ* and *FABP4*. In a previous study, *CyclinD1* and *CDK4* mainly affected fat deposition by constructing a Cyclin D/Cdk4 complex [36]. *PPARγ* and *FABP4* mainly affected fat deposition through the PPARγ pathway [37,38]. Thus, we inferred that the possible reasons why *NTN1* did not affect the expression of these factors could be either that *NTN1* played its role in a different pathway, or *NTN1* regulated fat deposition only by promoting muscle cell differentiation. In any case, the important mechanism of *NTN1* for myoblast and adipocyte proliferation and differentiation is worthy of further exploration.

## 5. Conclusions

The quantitative expression results of RNA and protein showed that the *NTN1* gene played an important role in the development of muscle or fat. Further studies have shown that *NTN1* could significantly regulate the proliferation and differentiation of C2C12 cells but have no effect on the proliferation and differentiation of 3T3-L1 preadipocytes. It is speculated that the copy number of the *NTN1* gene affects the expression of genes through the gene dose, which in turn affects the deposition of intramuscular fat by affecting muscle development through an AMPK-related pathway.

## Figures and Tables

**Table 1:** Primer sequences for RT-qPCR.

Gene Symbol *	Sense Strands (5′-3′)	Anti-Sense STRANDS (5′-3′)
*NTN1*	TTGCAAAGCCTGTGATTGCC	AATCTTGATGCAAGGGGCAG
*GCG*	AAGCTTCAAACAGGGGTACAAT	CCACTTGGAATGTTACCCTAATG
*GAPDH*	CACUCAAGAUUGUCAGCAATT	UUGCUGACAAUCUUGAGUGAG
*KI67*	AGCTAACTTGCGCTGACTGG	ATATTGCCTCTTGCTCTTTGACT
*P21*	GTACTTCCTCTGCCCTGCTGCA	CCAATCTGCGCTTGGAGTGATAG
*MYOD*	GGCAGAATGGCTACGACAC	GGGTCTGGGTTCCCTGTTCT
*MYOG*	CGGTGGAGGATATGTCTGTTG	GGTGTTAGCCTTATGTGAATGG
*MCK*	AGGAGTACCCAGACCTCAGCAA	GACCGTGTAGGACTCCTCATCG
*CyclinD1*	CAGTAACGTCACACGGACTACAGG	CGTTGAGGAGATTGGTGTCAGG
*PPAR* *γ*	AAGAGCTGACCCAATGGTTG	ACCCTTGCATCCTTCACAAG
*FABP4*	TAAAAACACCGAGATTTCCTTCA	CCTTTCATAACACATTCCACCA
*CDCK4*	CTACATACGCAACACCCG	TCAAAGATTTTCCCCAACT

* *NTN1*: netrin-1; *GCG*: glucagon; *GAPDH*: glyceraldehyde-3-phosphate dehydrogenase; *KI67*: antigen identified by monoclonal antibody Ki 67; *P21*: cyclin-dependent kinase inhibitor; *MYOD*: class I myosin; *MYOG*: myogenin; *MCK*: muscle creatine kinase; *CyclinD1*: cyclin domain 1; *PPARγ*: peroxisome proliferator-activated receptor gamma; *FABP4*: fatty acid binding protein 4; *CDCK4*: cyclin dependent kinase 4.

**Table 2:** si-RNA targeting the mouse *NTN1* coding region.

si-NTN1	Sense Strands (5′-3′)	Anti-Sense STRANDS (5′-3′)
si-1085	GCGCGACUCCUAUUACUAUTT	AUAGUAAUAGGAGUCGCGCTT
si-1225	GCGACCGUUGCAAGCCCUUTT	AAGGGCUUGCAACGGUCGCTT
si-562	GCAACUCUUCGGAUCCCAATT	UUGGGAUCCGAAGAGUUGCTT
NC	UUCUCCGAACGUGUCACGUTT	UUCUCCGAACGUGUCACGUTT
